# The brain in healing mode - the intelligence of sadness

**DOI:** 10.1192/j.eurpsy.2026.11454

**Published:** 2026-07-31

**Authors:** J. Hortet, S. Duarte, C. Matias, T. Vieira, F. Santos, P. Coelho, A. Silva, R. Lopes

**Affiliations:** Hospital, Tomar, Portugal

## Abstract

**Introduction:**

Sadness is a fundamental, evolutionarily conserved negative emotion associated with loss, frustration, and social disconnection. Although commonly interpreted as a marker of psychopathology, emerging evidence highlights its regulatory and potentially adaptive role. Neurobiological findings suggest that sadness facilitates cognitive reappraisal, motivational reorganization, and energy conservation, underscoring the need to distinguish functional sadness from maladaptive depressive states.

**Objectives:**

To critically analyze current evidence on the neurobiology of sadness, integrating findings from neuroimaging, psychophysiology, and theoretical models of emotion, and to clarify its adaptive functions and clinical implications.

**Methods:**

A narrative and integrative review of literature published between 2015 and 2024 was conducted, encompassing systematic reviews, meta-analyses, and experimental studies.

**Results:**

Convergent findings reveal activation of a distributed neural circuit involving the subgenual anterior cingulate cortex, amygdala, insula, and periaqueductal gray, modulated by medial and dorsolateral prefrontal regions. Frontal asymmetry and parasympathetic predominance are consistent with behavioral withdrawal and self-regulation states. At adaptive levels, sadness promotes emotional integration and behavioral reorientation; when prolonged or rigid, it may evolve into maladaptive affective dysregulation.

**Conclusions:**

Conceptualizing sadness as a neurobiologically adaptive process reframes its clinical interpretation, emphasizing emotional function and regulatory flexibility. This perspective may inform psychotherapeutic and psychiatric interventions aimed at restoring affective plasticity rather than suppressing negative emotion.

**Disclosure of Interest:**

None Declared

